# Radiofrequency Ablation In Ventricular Fibrillation

**Published:** 2008-11-01

**Authors:** Jayachandran Thejus, Johnson Francis, Michel Haissaguerre

**Affiliations:** 1 Calicut Medical College, Kerala, India; 2 Universite Bordeaux, Hopital Haut-Leveque, Bordeaux-Pessac, France

**Keywords:** ventricular fibrillation, radiofrequency catheter ablation

Ventricular fibrillation (VF) is the most common arrhythmic cause of sudden cardiac death. The present recommendation for prevention of VF is ICD implantation. However, ICDs are costly; the shocks they give are often very uncomfortable for the patient and they may need reimplantation if there is battery depletion. Radiofrequency ablation of VF was pioneered by Haissaguerre [1] and is presently being tried by many investigators.

VF is triggered mainly by an ectopic impulse falling in the "vulnerable" period and it is maintained by multiple reentrant wavelets. RFA of VF aims at controlling the trigger, that is, to eliminate the ectopic focus.

## Idiopathic VF

Idiopathic VF is a VF occurring without structural heart disease or ECG abnormalities. It is precipitated mainly by ventricular premature complexes (VPCs) from the Purkinje system. On reviewing 34 reported cases [1-8], it was found that 27 cases (79 %) were from the Purkinje system, 6 cases were from the right ventricular outflow tract (RVOT)  and 1 case, reported by Tanimoto et al [8],  was from the tricuspid annular region of the right ventricle (not from the overlying Purkinje fibers).

The optimal timing for the mapping/RFA procedure is when the patient has frequent VPCs. The origin of the VPCs is localized by mapping the earliest site of electrical activity (if there is no spontaneous VPC, pace mapping is needed). Purkinje origin is identified by the Purkinje potential occurring before the VPC. The Purkinje potential is a sharp spike of less than 10 msec duration. If the VPC is occurring without a Purkinje potential, it means that it has a muscular origin.

In Purkinje VPC leading to VF, compared to RVOT VPC leading to VF, there are fewer VPCs, but these VPCs are more likely to cause VF. The former is more likely to be multifocal, necessitating more time for RFA compared to the latter, which is usually unifocal. The site of origin of the VPCs is ablated till VPCs cease to occur. In the case of Purkinje VPC, abolition of local Purkinje potential is another procedural endpoint. At the time of ablation, usually there is a transient exacerbation of VPCs, which can rarely cause a VF.

The VPCs could be successfully ablated in all reported cases. There is a low chance of recurrence (of about 9 % over a mean follow up period of 22 months). This is certainly due to the limited and endocardial location of the targeted tissue.

## VF in post myocardial infarction patients

The site of origin of the VPCs causing VF in post MI patients is the Purkinje network in the border zone of the infarct. After MI, the Purkinje network is only partially ischemic as it is nourished by blood in the ventricular cavity. This makes it electrically unstable, leading to VPCs and subsequently VF.

Usually VF in post MI patients is medically manageable and not recurrent. Only rarely is it recurrent and drug refractory necessitating RFA. The ectopic foci in the Purkinje system can usually be readily localized and ablated. RFA was successful in all the reported 17 cases [9-13]. There was a low recurrence rate of 6% over a mean follow up of 10 months.

Very recently, a case was reported, by Takahashi et al [12], in which VF in a post MI patient occurred from VPCs from a focus in the right ventricular myocardium (not from the Purkinje network). The focus could be successfully ablated and there was no recurrence over 4 months.

## Brugada syndrome causing VF

In Brugada syndrome, VF is caused mainly by VPCs from the RVOT. Ablation is highly successful and durable. In 4 out of 5 cases of RFA reported for Brugada syndrome, the VPCs were from the RVOT, while in the remaining one case, it was from the Purkinje network [14-16]. The VPCs could be successfully ablated in all cases and there was no recurrence on follow up (mean 17 months).

## Long QT syndrome leading to VF

In LQTS leading to VF, out of 4 reported cases, in 2 cases the VPCs were from the Purkinje network, in 1 case it was from the posterior fascicle and in 1 case it was from the RVOT [16]. In all cases ablation was successful and there was no recurrence on follow up (mean 17 months).

## Catecholaminergic Polymorhpic Ventricular Tachycardia (CPVT) causing VF

RFA was successfully done in a case of CPVT with VF, the site of origin of VPCs being the Purkinje network. On follow up, there was no recurrence of VF (mean 2 yrs) [17].

## Other causes of VF

Sanders [13] reported a case, in 2004, in which a girl with an unclassified left ventricular repolarization syndrome with recurrent VF was subjected to EPS with the intent of doing RFA, but due to inability to map due to frequent multiple VPCs, RFA could not be done. Two cases of infiltrative amyloidosis of the heart leading to VF were subjected to RFA by Mlcochova. [18] In one case, the site of origin of the culprit VPCs was the Purkinje network while in the other, it was the posterior fascicle. In both cases, t­­­­­­he trigger could be ablated, with no recurrence on follow up. Li [19] reported RFA for VF after aortic valve repair, while Sanders [13] reported RFA for VF after aortic valve replacement. In both cases, the sites of origin of the inciting VPCs were in the Purkinje network and could be successfully ablated.

## Conclusions

Thus, it can be seen that in most cases, with careful technique, VF can be safely ablated. In most cases, there is no recurrence of VF, even on long term follow up. Successful RFA for VF decreases the frequency of ICD shocks, thus improving the quality of life of the patient and also the longevity of the ICD. Even though presently RFA is done in VF mainly to decrease the frequency of ICD shocks, future perfections of the technique may obviate the need for an ICD altogether.
